# Pharmacokinetics of Gepotidacin in Subjects With Normal Hepatic Function and Hepatic Impairment

**DOI:** 10.1002/cpdd.913

**Published:** 2021-01-15

**Authors:** Mohammad Hossain, Courtney Tiffany, Yu Tao, Aline Barth, Thomas C. Marbury, Richard A. Preston, Etienne Dumont

**Affiliations:** ^1^ GlaxoSmithKline Collegeville Pennsylvania USA; ^2^ Orlando Clinical Research Center Orlando Florida USA; ^3^ Clinical Pharmacology Research Unit Division of Clinical Pharmacology University of Miami Department of Medicine Miller School of Medicine University of Miami Clinical and Translational Science Institutes (CTSI) and The Peggy and Harold Katz Family Drug Discovery Center Miami Florida USA

**Keywords:** gepotidacin, liver failure, pharmacokinetics, safety

## Abstract

Gepotidacin is a novel triazaacenaphthylene bacterial topoisomerase inhibitor. This phase 1 nonrandomized, open‐label, multicenter, 2‐part study evaluated the pharmacokinetics, safety, and tolerability of oral gepotidacin 1500 mg in 3 different hepatic settings (normal, moderate impairment, and severe impairment). Gepotidacin was safe and generally tolerated in all subjects. Compared to subjects with normal hepatic function, gepotidacin plasma area under the plasma concentration–time curve from time 0 to infinity (AUC_0–∞_) and maximum concentration significantly increased by 1.7‐ and 1.9‐fold, respectively, in severe hepatic impairment; increases in moderate impairment were not statistically significant. No significant effect was observed for gepotidacin plasma elimination half‐life (geometric mean range, 8.2–9.1 hours) across hepatic groups. Renal clearance increased in moderate (16%) and severe (52%) hepatic impairment vs normal. The mean fraction of gepotidacin dose excreted in urine increased with increasing hepatic impairment (normal, 7.5%; moderate, 11.2%; and severe, 19.9%). Urine gepotidacin concentrations remained high for 12 hours in all hepatic groups after dosing. Saliva gepotidacin concentrations displayed a linear relationship with plasma concentrations (R^2^ = 0.76). The ratio of saliva AUC to unbound plasma AUC and elimination half‐life were not affected by hepatic impairment. These data indicate that gepotidacin dose adjustment is not required in mild to moderate hepatic impairment; severe hepatic impairment may require increases in dosing interval or dose reduction.

Gepotidacin is a novel triazaacenaphthylene bacterial topoisomerase inhibitor that is being developed for the treatment of urogenital gonorrhea and uncomplicated urinary tract infection. Gepotidacin selectively inhibits bacterial DNA replication by interacting in a unique way on the GyrA subunit of bacterial DNA gyrase and the ParC subunit of bacterial topoisomerase IV.^1^, ^2^, ^3^, ^4^ This interaction appears to be highly specific to bacterial topoisomerases, as evidenced by weak in vitro inhibition of human topoisomerase IIα, supporting the selective activity of gepotidacin against the bacterial target. The novel mode of action of this new class of antibacterial affords in vitro activity against target pathogens resistant to established antibacterials, including fluoroquinolones. In clinical trials, gepotidacin has demonstrated efficacy in acute bacterial skin and skin structure infections, uncomplicated urogenital gonorrhea, and uncomplicated urinary tract infection.^5^, ^6^, ^7^, ^8^ Gepotidacin is currently in phase 3 clinical studies for the treatment of uncomplicated urinary tract infection (1500 mg twice daily for 5 days) (ClinicalTrials.gov identification numbers NCT04020341 and NCT04187144) and uncomplicated urogenital gonorrhea (2 doses of 3000 mg separated by 10–12 hours) (ClinicalTrials.gov identification number NCT04010539).

In a study with healthy subjects (ClinicalTrials.gov identification number NCT02202187), after fasted administration of single oral doses of gepotidacin capsules from 100 to 3000 mg, the median time to reach maximum concentrations (t_max_) ranged from 1.0 to 3.5 hours with mean elimination half‐life (t_1/2_) values ranging from 12 to 19 hours (100‐mg dose excluded).^9^ Values for area under the concentration‐time curve from time 0 to infinity (AUC_0–∞_) and from time 0 to last quantifiable concentration (AUC_0–t_), and maximum observed concentration (C_max_) increased in a greater‐than‐dose‐proportional manner over the dose range, with a trend toward dose proportionality when the low dose of 100 mg was excluded. In a previous absorption, distribution, metabolism, and excretion study for gepotidacin, the mean recovery of radioactivity in urine and feces accounted for approximately 31% and 53%, respectively, of [^14^C]‐gepotidacin administered as a single oral dose.^4^ After oral administration, gepotidacin was eliminated mainly as parent in urine, accounting for approximately 20% of the administered dose. Elimination via metabolism (urine plus feces) accounted for a total of 13% of the dose. Biliary and renal clearances (CL_r_) were 16.9% and 43.9%, respectively. The clinically observed total intravenous clearance was approximately 43 L/h across several doses and CL_r_ was approximately 16 L/h across 2 doses.^10^ Together, these data indicate a hepatic clearance of 27 L/h, suggesting that hepatic clearance is a major route of elimination of gepotidacin.

The oxidative metabolism of gepotidacin is mediated primarily by cytochrome P450 enzyme 3A4.^4^ In a clinical drug‐drug interaction study, when itraconazole (inhibitor of both P‐glycoprotein and cytochrome P450 3A4) was coadministered with gepotidacin, a weak drug‐drug interaction (40% increase in C_max_ and 50% increase in AUC_0–∞_) was observed.^11^ In patients with severe renal impairment (with and without dialysis), gepotidacin dosing at 750 mg resulted in significant increases in plasma drug levels and decreases in clearance with minimal impact on t_1/2_.^12^ Alpha‐1‐acid glycoprotein (AAG) plays a greater role in plasma protein binding of strongly basic compounds^13^ such as gepotidacin. Protein binding was determined in human plasma samples using equilibrium dialysis. There was no significant difference in protein binding of gepotidacin among low (32.2%), mid (33%), and high (29%) AAG plasma groups (data on file). The protein binding of gepotidacin at the mean AAG concentration (33%) was used in the human free fraction calculation of gepotidacin.

The current study was designed to describe the pharmacokinetics (PK) of gepotidacin in blood, saliva, and urine in subjects with and without hepatic impairment.

## Subjects and Methods

### Study Population

Eligibility criteria included male or female (nonpregnant, nonlactating) subjects between 18 and 80 years of age, inclusive. Part 1 included participants with normal hepatic function or with moderate hepatic impairment. Part 2 included participants with normal hepatic function or severe hepatic impairment. Healthy subjects were in clinically stable health as determined by the investigator based on medical history, clinical laboratory results (serum chemistry, hematology, urinalysis, and serology), vital sign measurements, 12‐lead electrocardiogram (ECG) results, and physical examination findings. Hepatic impairment was defined according to the Child–Pugh classification system defined in the Food and Drug Administration (FDA) Guidance for Industry, Pharmacokinetics in Patients with Impaired Hepatic Function as follows: Child–Pugh score of 5 to 6 (mild hepatic impairment), 7 to 9 (moderate hepatic impairment), or 10 to 15 (severe hepatic impairment). Hepatically impaired participants had known medical history of liver disease (with or without history of alcohol abuse) and previous confirmation of liver cirrhosis by liver biopsy or other medical imaging technique associated with unambiguous medical history. Participants with hepatic impairment were allowed to be on a stable regimen of chronic medications (for at least 7 days before dosing until completion of the follow‐up visit) if, in the opinion of the investigator, the medications would not interfere with the conduct of the study. In addition, hepatically impaired participants had platelet counts ≥30,000 × 10^9^/L of blood, with no major bleeding episodes within the past 6 months. Participants with chronic hepatitis B or C (duration >6 months) were eligible for enrollment.

This study was conducted between June 2018 and December 2018 at 2 centers in the United States—Orlando Clinical Research Center (Orlando, Florida) and the University of Miami, Division of Clinical Pharmacology (Miami, Florida)—according to the ethical principles of Good Clinical Practice and the Declaration of Helsinki after a written informed consent was obtained from each subject. The protocol and the informed consent were approved by IntegReview Institutional Review Board (Austin, Texas) and University of Miami Human Subject Research Office (Miami, Florida).

### Study Design

This was a phase 1, nonrandomized, open‐label, multicenter, 2‐part study that evaluated the PK, safety, and tolerability of a single 1500‐mg oral dose of gepotidacin (2 × 750 mg tablets with food, ∼30 minutes after receiving a standard breakfast) in participants with varying degrees of hepatic impairment matched to healthy participants with normal hepatic function in terms of gender distribution, age (approximately ±10 years), and body mass index (approximately ±20%).

Part 1 included participants with normal hepatic function and moderate hepatic impairment. Based on the PK, safety, and tolerability data of Part 1, Part 2 enrolled participants with severe hepatic impairment and subsequently enrolled participants with normal hepatic function to match those with severe hepatic impairment. Because Part 1 did not indicate clinically relevant differences between participants with normal hepatic function versus those with moderate hepatic impairment, Part 2 excluded participants with mild hepatic impairment.

### Pharmacokinetic Assessments and Analysis

For all subjects, serial blood samples for PK analysis of gepotidacin were collected at the following time points: 0, 0.5, 1, 1.5, 2, 2.5, 3, 4, 6, 8, 12, 24, 36, and 48 hours after dosing.

Urine collection for subjects with normal hepatic function occurred at the following time intervals: 0 (before dosing), 0 to 2, 2 to 4, 4 to 6, 6 to 8, 8 to 12, 12 to 24, 24 to 36, and 36 to 48 hours. Urine collection intervals for subjects with hepatic impairment included predose, 0 to 6, 6 to 12, 12 to 24, 24 to 36, and 36 to 48 hours.

Saliva samples were collected for PK analysis of gepotidacin at the following times: before dosing, 0.5, 1, 1.5, 2, 2.5, 3, 4, 6, 8, 12, 24, 36, and 48 hours.

Concentrations of gepotidacin were determined in plasma, urine, and saliva using validated bioanalytical methodologies at PPD Laboratories (Middleton, Wisconsin) as previously described.^11^ All samples were shipped frozen on dry ice and frozen at −20°C upon arrival.

### Safety Assessments

Safety assessments were conducted at baseline, during the dosing periods, and at the follow‐up visit and included the following: adverse events (AEs), clinical laboratory evaluations (chemistry, hematology, and urinalysis), pregnancy tests, vital signs, and 12‐lead ECGs. These data were descriptively analyzed.

### Statistical Analyses

PK parameters were estimated following a single oral dose of gepotidacin in subjects with moderate and severe hepatic impairment and compared with the PK parameters from matched subjects with normal hepatic function.

The sample size was considered sufficient to determine meaningful differences between the PK parameters in normal vs hepatically impaired subjects. The PK parameters were derived using standard noncompartmental methods using Phoenix WinNonlin version 6.4 (Certara, Princeton, New Jersey). All PK end points were prospectively defined before analysis. Descriptive statistics were summarized for demographic variables. Plasma, urine, and saliva concentrations for all subjects and the associated PK parameters were summarized statistically.

The natural log‐transformed plasma and saliva AUC_0–∞_ and C_max_ values for gepotidacin in the hepatic impairment groups and normal hepatic function group were compared using an analysis of variance (ANOVA). The natural log‐transformed urine AUC from time 0 to 48 hours and nontransformed CL_r_ values for gepotidacin in the hepatic impairment groups and normal hepatic function group were also compared using ANOVA. T_max_ of gepotidacin was analyzed using the nonparametric method.^14^

## Results

### Demographics and Disposition

A total of 25 participants were enrolled in the study (Table 1). In Part 1, 8 participants with normal hepatic function were matched to 8 participants with moderate hepatic impairment. In Part 2, 8 participants with severe hepatic impairment were matched to 7 participants with normal hepatic function who participated in Part 1 and 1 additional participant with normal hepatic function.

**Table 1:** Summary of Demographics and Baseline Characteristics

Demographics	Normal Hepatic Function (N = 9)	Moderate Hepatic Impairment (N = 8)	Severe Hepatic Impairment (N = 8)	Total (N = 25)
Age, y,^a^ mean (SD)	59.8 (5.7)	62.5 (7.2)	58.1 (6.2)	60.1 (6.3)
Age ranges,^a^ n (%)				
Adult, 18–64 y	7 (77.8)	5 (62.5)	6 (75.0)	18 (72.0)
≥65–84 y	2 (22.2)	3 (37.5)	2 (25.0)	7 (28.0)
Sex, n (%)				
Female	1 (11.1)	1 (12.5)	0	2 (8.0)
Male	8 (88.9)	7 (87.5)	8 (100)	23 (92.0)
BMI, kg/m^2^, mean (SD)	29.79 (3.49)	32.75 (4.34)	29.59 (4.41)	30.67 (4.17)
Height, cm, mean (SD)	171.50 (6.78)	173.28 (10.36)	175.08 (6.06)	173.21 (7.72)
Weight, kg, mean (SD)	87.46 (10.02)	98.55 (17.87)	91.20 (17.21)	92.20 (15.34)
Ethnicity, n (%)				
Hispanic or Latino	5 (55.6)	4 (50.0)	4 (50.0)	13 (52.0)
Not Hispanic or Latino	4 (44.4)	4 (50.0)	4 (50.0)	12 (48.0)
Race, n (%)				
Black/African heritage	3 (33.3)	0	0	3 (12.0)
Asian–Central/South Asian heritage	0	0	1 (12.5)	1 (4.0)
White–White/Caucasian/European heritage	6 (66.7)	8 (100.0)	7 (87.5)	21 (84.0)
Child–Pugh total score, n (%)				
7	NA	3 (37.5)	0	NA
8	NA	4 (50.0)	0	NA
9	NA	1 (12.5)	0	NA
10	NA	0	4 (50.0)	NA
11	NA	0	4 (50.0)	NA
Liver disease–related medical conditions, n (%)		7 (87.5)	8 (100)	15 (60.0)
Alcoholic liver disease	0	3 (37.5)	6 (75.0)	9 (36.0)
Chronic hepatitis C	0	3 (37.5)	5 (62.5)	8 (32.0)
Chronic hepatitis B	0	0	1 (12.5)	1 (4.0)
Nonalcoholic steatohepatitis	0	1 (12.5)	0	1 (4.0)

BMI, body mass index; NA, not applicable; SD, standard deviation.

^a^ Age was imputed when full date of birth was not provided.

### Pharmacokinetics

#### Pharmacokinetics in plasma

**Figure 1:**
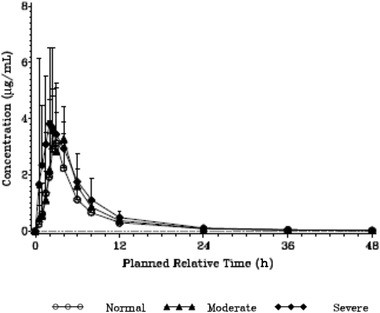
Mean gepotidacin plasma concentrations. Dashed line represents lower limit of quantification of 0.01 μg/mL. Values below the lower limit of quantification were set to 0 and included in the calculation of arithmetic mean.

Following single oral administration of 1500 mg of gepotidacin in participants with moderate and severe hepatic impairment, peak concentration (C_max_) to gepotidacin in plasma was attained (t_max_) at a median of 2.75 and 2.25 hours after dosing, respectively. A similar t_max_ was attained at a median 3.00 hours after dosing in matched healthy controls (Table 2, Figure 1). Geometric mean apparent clearance (CL/F) progressively decreased in participants with increasing hepatic impairment relative to normal hepatic function. There was no substantial change in t_1/2_ with increasing hepatic impairment, with geometric mean t_1/2_ values of 8.5, 8.2, and 9.1 hours observed for the moderate, severe, and normal hepatic groups, respectively. The percent coefficient of variation for the geometric means between subjects (%CVb) in the extent of systemic exposure to gepotidacin (C_max_, AUC over the dosing interval [AUC_0–t_], and AUC_0–∞_) was generally comparable within each parameter and across all hepatic groups. The %CVb values ranged from 42.7% to 85.0% for C_max_ and from 30.1% to 45.8% for AUC_0–t_ and AUC_0–∞_.

**Table 2:** Summary of Gepotidacin Plasma Pharmacokinetic Parameters by Group

Parameter		Normal Hepatic Function (N = 9)	Moderate Hepatic Impairment (N = 8)	Severe Hepatic Impairment (N = 8)
AUC_0–t_, μg • h/mL	Geometric mean (%CVb)	15.5 (45.8)	19.2 (43.4)	25.1 (30.5)
	Arithmetic mean (SD)	16.8 (7.00)	20.6 (8.14)	26.1 (7.87)
AUC_0–∞_, μg • h/mL	Geometric mean (%CVb)	15.9 (44.1)	19.5 (42.6)	25.4 (30.1)
	Arithmetic mean (SD)	17.2 (6.99)	20.9 (8.22)	26.4 (7.85)
C_max_, μg/mL	Geometric mean (%CVb)	3.20 (85.0)	3.91 (64.1)	5.54 (42.7)
	Arithmetic mean (SD)	3.85 (1.99)	4.49 (2.30)	6.03 (3.02)
t_max_, h	Median (minimum–maximum)	3.00 (1.50, 6.00)	2.75 (2.50, 4.00)	2.25 (0.50, 4.00)
t_lag_, h	Median (minimum–maximum)	0.50 (0.0, 2.5)	0.00 (0.0, 1.0)	0.00 (0.0, 1.0)
t_1/2_, h	Geometric mean (%CVb)	9.07 (14.9)	8.52 (12.3)	8.21 (15.2)
	Arithmetic mean (SD)	9.16 (1.41)	8.57 (1.06)	8.29 (1.28)
CL/F, L/h	Geometric mean (%CVb)	94.4 (44.1)	76.9 (42.6)	59.0 (30.1)
	Arithmetic mean (SD)	102 (43.7)	82.8 (35.2)	61.3 (17.7)

%CVb, percent of coefficient of variation between subjects; AUC_0–∞_, area under the concentration‐time curve from time 0 to infinity; AUC_0–t_, area under the concentration‐time curve from time 0 to the time of the last quantifiable concentration; CL/F, apparent oral clearance; C_max_, maximum observed concentration; t_1/2_, terminal phase half‐life; t_lag_, lag time before observation of drug concentrations; t_max_, time to first occurrence of C_max_.

Statistical analysis of gepotidacin log_10_‐transformed primary plasma PK parameters was performed using an ANOVA model (Table S1). Compared with matched healthy controls, C_max_ and AUC_0–∞_ were approximately 1.2‐fold higher in participants with moderate hepatic impariment. In severe hepatic impairment, AUC_0–∞_ and C_max_ were significantly greater than in matched healthy controls (1.7‐fold and 1.9‐fold greater, respectively). T_max_ was generally similar across the treatment groups (Table S2).

#### Pharmacokinetics in urine

**Figure 2:**
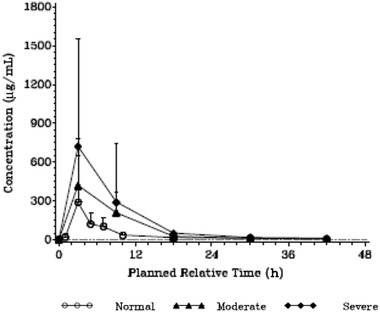
Mean gepotidacin urine concentrations. Dashed line represents lower limit of quantification of 1.00 μg/mL. Values below the lower limit of quantification were set to 0 and included in the calculation of arithmetic mean.

Median urine concentrations for gepotidacin following single oral administration of 1500 mg of gepotidacin were generally higher over the first 24 hours for the moderate hepatic group and across the entire time profile for the severe hepatic group compared with that observed in healthy matched controls (normal) (Table 3, Figure 2). Following single oral administration of 1500 mg of gepotidacin in participants with moderate and severe hepatic impairment, gepotidacin urinary exposure (AUCs), CL_r_, and amount excreted in urine were higher than that observed in matched healthy controls (normal) and increased with hepatic impairment severity (Table 3).

**Table 3:** Summary of Gepotidacin Urine Pharmacokinetic Parameters by Group

Parameter		Normal Hepatic Function (N = 9)	Moderate Hepatic Impairment (N = 8)	Severe Hepatic Impairment (N = 8)
AUC_0–12_, μg • h/mL	Geometric mean (%CVb)	832 (118)	2164 (131)	3285 (110)
	Arithmetic mean (SD)	1201 (1034)	3030 (2246)	4771 (4678)
AUC_0–24_, μg • h/mL	Geometric mean (%CVb)	938 (105)	2274 (105)	4247 (99.1)
	Arithmetic mean (SD)	1269 (1040)	2925 (1868)	5807 (5151)
AUC_0–48_, μg • h/mL	Geometric mean (%CVb)	991 (114)	3162 (61.9)	3902 (81.3)
	Arithmetic mean (SD)	1394 (1252)	3547 (1668)	5035 (4696)
CL_r_, L/h	Geometric mean (%CVb)	7.59 (46.6)	9.08 (32.8)	11.8 (38.7)
	Arithmetic mean (SD)	8.18 (3.21)	9.45 (2.71)	12.5 (3.91)
fe%, %	Geometric mean (%CVb)	7.53 (61.9)	11.2 (70.9)	19.9 (52.2)
	Arithmetic mean (SD)	8.51 (4.24)	12.8 (5.63)	21.7 (8.29)
Ae total, mg	Geometric mean (%CVb)	113 (61.9)	168 (70.9)	299 (52.2)
	Arithmetic mean (SD)	128 (63.6)	191 (84.4)	325 (124)

%CVb, percent of coefficient of variation between subjects; Ae total, total unchanged drug; AUC_0–12,_ area under the concentration‐time curve from time 0 to 12 hours; AUC_0–24_, area under the concentration‐time curve from time 0 to 24 hours; AUC_0–48_, area under the concentration‐time curve from time 0 to 48 hours; AUC_0–t_, area under the concentration‐time curve from time 0 to the time of the last quantifiable concentration; CL_r_, renal oral clearance; fe%, percentage of the given dose of drug excreted in urine; t_max_, time to first occurrence of C_max_.

In participants with normal hepatic function, 7.5% of the total gepotidacin dose was removed in the urine, which was increased to 11.2% and 19.9% for the moderate and severe hepatic impairment groups, respectively (geometric mean estimates).

Statistical analysis of gepotidacin log_10_‐transformed urine primary PK parameters was performed using an ANOVA model (Supplementary Table S3). Following single oral administration of 1500 mg of gepotidacin in participants with moderate and severe hepatic impairment, overall urinary exposure (AUC from time 0 to 48 hours) to gepotidacin was approximately 3.2‐fold and 3.9‐fold greater than that observed in matched healthy controls, respectively, which trended upward similar to increases observed in CL_r_ for the moderate and severe hepatic groups compared to the normal group (% increases estimates from the least squares means difference relative to normal).

#### Pharmacokinetics in saliva

**Figure 3:**
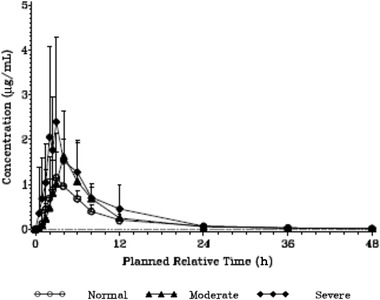
Mean gepotidacin saliva concentrations. Dashed line represents lower limit of quantification of 0.001 μg mL. Values below the lower limit of quantification were set to 0 and included in the calculation of arithmetic mean.

Following single oral administration of 1500 mg of gepotidacin in participants with moderate and severe hepatic impairment, peak concentration of gepotidacin in saliva was attained at ∼3.92 and 3.00 hours after dosing, respectively, which was generally comparable with 3.00 hours of matched healthy controls (median estimates) (Table 4, Figure 3). In line with the plasma data, geometric mean CL/F based on saliva exposure progressively decreased in participants with increasing hepatic impairment severity relative to normal hepatic function. There was no substantial change in t_1/2_ with increasing hepatic impairment, with geometric mean t_1/2_ values of 6.8, 7.2, and 9.2 hours observed for the moderate, severe, and normal hepatic groups, respectively (Table 4). The %CVb in the extent of saliva exposure to gepotidacin (C_max_, AUC_0–t_, and AUC_0–∞_) was generally comparable within each parameter and across all hepatic groups, with %CVb values ranging from 40.3% to 65.3%.

**Table 4:** Summary of Gepotidacin Saliva Pharmacokinetic Parameters by Group

Parameter		Normal Hepatic Function (N = 9)	Moderate Hepatic Impairment (N = 8)	Severe Hepatic Impairment (N = 8)
AUC_0–t_, μg • h/mL	Geometric mean (%CVb)	7.75 (42.3)	9.82 (47.6)	14.1 (47.6)
	Arithmetic mean (SD)	8.29 (3.02)	10.7 (4.41)	15.4 (7.10)
AUC_0–∞_, μg • h/mL	Geometric mean (%CVb)	8.04 (40.3)	9.53 (45.8)	14.3 (46.7)
	Arithmetic mean (SD)	8.54 (2.95)	10.2 (3.82)	15.6 (7.05)
C_max_, μg/mL	Geometric mean (%CVb)	1.29 (65.3)	1.65 (51.8)	2.70 (60.3)
	Arithmetic mean (SD)	1.52 (0.982)	1.83 (0.935)	3.11 (1.830)
t_max_, h	Median (minimum–maximum)	3.00 (1.92, 6.00)	3.92 (2.42, 8.00)	3.00 (1.92, 6.00)
t_lag_, h	Median (minimum–maximum)	0.500 (0.00, 1.50)	0.00 (0.00, 1.50)	0.00 (0.00, 0.917)
t_1/2_, h	Geometric mean (%CVb)	9.24 (47.4)	6.75 (19.6)	7.18 (33.6)
	Arithmetic mean (SD)	10.1 (4.52)	6.85 (1.23)	7.53 (2.48)
CL/F, L/h	Geometric mean (%CVb)	187 (40.3)	157 (45.8)	105 (46.7)
	Arithmetic mean (SD)	201 (91.7)	172 (88.3)	114 (51.6)
R_AUC0–t_ (ratio)	Geometric mean (%CVb)	0.746 (49.4)	0.765 (21.1)	0.839 (27.5)
	Arithmetic mean (SD)	0.818 (0.358)	0.780 (0.162)	0.866 (0.242)
R_AUC0–∞_ (ratio)	Geometric mean (%CVb)	0.755 (49.2)	0.765 (24.4)	0.838 (26.9)
	Arithmetic mean (SD)	0.828 (0.361)	0.784 (0.187)	0.865 (0.236)

%CVb, percent of coefficient of variation between subjects; AUC_0–∞_, area under the concentration‐time curve from time 0 to infinity; AUC_0–t_, area under the concentration‐time curve from time 0 to the time of the last quantifiable concentration; CL/F, apparent oral clearance; C_max_, maximum observed concentration; N, number of participants in the hepatic function group; R_AUC0–∞_, ratio of the AUC_0–∞_ observed in saliva relative to the unbound AUC_0–∞_ in plasma; R_AUC0–t_, ratio of the AUC_0–t_ observed in saliva relative to the unbound AUC_0–t_ in plasma; t_1/2_, terminal phase half‐life; t_lag_, lag time before observation of drug concentrations; t_max_, time to first occurrence of C_max_.

Statistical analysis of gepotidacin log_10_‐transformed primary saliva PK parameters was performed using an ANOVA model (Table S4). In participants with moderate and severe hepatic impairment, peak and overall exposure to gepotidacin (C_max_ and AUC_0–∞_) in saliva was 1.3‐fold and 1.2‐fold, and 2.1‐fold and 1.8‐fold greater than that observed in participants with normal hepatic function, respectively. No clinically meaningful differences in saliva t_max_ was observed between the participants with hepatic impairment and those with normal hepatic function (Table S5).

**Figure 4:**
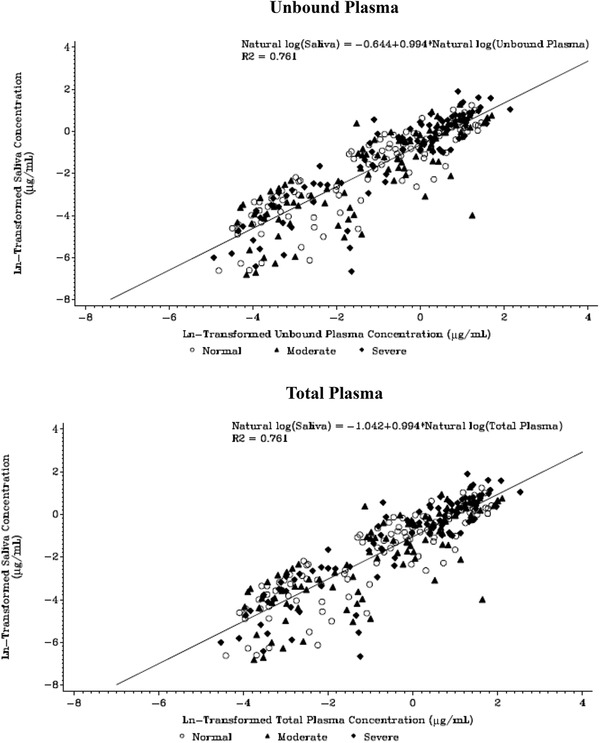
Scatter plot of gepotidacin saliva and unbound and total plasma concentrations by hepatic function group. Lower limit of quantification = 10.0 ng/mL; a correction factor of 0.67 was applied to the total plasma concentrations to derive unbound values. Moderate hepatic impairment: Child–Pugh score 7–9. Severe hepatic impairment: Child–Pugh score 10–15.

Gepotidacin concentrations in saliva correlated well with unbound and total plasma gepotidacin concentrations in a positive manner, with an R^2^ value of 0.76 (Figure 4).

### Safety

Overall, there were no safety findings of potential clinical concern in subjects with moderate or severe hepatic impairment (Table 5). All but 1 AE was mild in severity and considered related to gepotidacin by the investigator. Gastrointestinal events were the most commonly reported system organ class of AEs. All AEs resolved by the end of the study, with the exception of 1 participant with an unrelated moderate wrist fracture 4 days after gepotidacin dosing and 1 participant with normal hepatic function who had a fatal serious AE of nocturnal death that was not considered drug related. One participant with severe hepatic impairment experienced an AE of mild leukopenia 8 days after dosing. The AE resolved approximately 3 days later without treatment and was considered related to gepotidacin. There were no other clinically important changes in clinical laboratory parameters, vital signs, or ECGs during this study (data not shown).

**Table 5:** Summary of All Adverse Events

Preferred Term, n (%)	Normal Hepatic Function (N = 9)	Moderate Hepatic Impairment (N = 8)	Severe Hepatic Impairment (N = 8)	Total (N = 25)
Any event	4 (44.4)	2 (25.0)	4 (50.0)	10 (40.0)
Diarrhea	3 (33.3)	2 (25.0)	2 (25.0)	7 (28.0)
Headache	0	1 (12.5)	1 (12.5)	2 (8.0)
Nausea	0	0	1 (12.5)	1 (4.0)
Leukopenia	0	0	1 (12.5)	1 (4.0)
Death	1 (11.1)	0	0	1 (4.0)
Wrist fracture	0	1 (12.5)	0	1 (4.0)
Decreased appetite	0	0	1 (12.5)	1 (4.0)

## Discussion

Review of data from Part 1 of the study, which included participants with normal liver function and those with moderate hepatic impairment, showed that the safety and PK requirements were met and that it was appropriate to proceed with Part 2 of the study in participants with severe hepatic impairment. Participants with mild hepatic impairment were not evaluated since the difference in PK between participants with moderate hepatic impairment compared to participants with normal hepatic function were not statistically significant and not considered to be clinically relevant (1.2‐fold increase in plasma AUCs and C_max_ in the moderate hepatic impaired group). Therefore, dose adjustment in participants with moderate or mild hepatic impairment is not considered necessary.

In participants with severe hepatic impairment (Part 2), gepotidacin C_max_ and AUC_0–∞_ significantly increased by approximately 1.7‐fold and 1.9‐fold, respectively, compared to those with normal hepatic function. The increase in exposure is due to a decrease in hepatic clearance that also caused a reduction in the overall drug clearance (CL/F). These data suggest that an increase in the dosing interval or dose reduction may be necessary for participants with severe hepatic impairment.

Normal hepatic function participants had 7.5% of the drug extracted in urine, while moderate and severe hepatic impairment participants had approximately 11.2% and 19.9% removed in urine, respectively. Similarly, the geometric mean CL_r_ was increased by approximately 16% and 52% in participants with moderate and severe hepatic impairment, respectively, compared with that observed in matched healthy controls (percent increases estimated from the least squares means difference relative to normal). This observation can be possibly due to decreased protein binding in hepatic impairment given that there could be a decrease in protein production in the liver that could result in the availability of more unbound drug for renal clearance.

The presence of high urine concentrations would be relevant in the treatment of urinary tract infections. Efficacy of gepotidacin was demonstrated in a phase 2 study in participants with uncomplicated urinary tract infection, in which the minimum gepotidacin urine concentrations remained above the clinically relevant gepotidacin minimum inhibitory concentration value of 4 μg/mL throughout the dosing interval.^8^

Saliva concentrations displayed a linear relationship with plasma (both bound and unbound) gepotidacin concentrations (R^2^ = 0.76). The ratio of saliva AUC to unbound plasma AUC was consistently close to unity (ratio of the AUC_0–t_ and AUC_0–∞_ ranged from 0.746 to 0.839). Saliva PK parameters (AUC_0–∞_, C_max_, and CL/F) also displayed a linear relationship with respect to total and unbound plasma PK parameters (AUC_0–∞_, C_max_, and CL/F); however, the trend did not lead to a high correlation coefficient (R^2^ = 0.511‐0.512) for saliva and plasma concentration relationship, possibly due to the small sample size. The good correlation between saliva and plasma free drug concentrations indicate that saliva may be used as a surrogate for measuring gepotidacin PK when there are limitations to collecting plasma samples (eg, in pediatrics).

It should be noted that urine and saliva drug concentrations are considered to be unbound drug and do not require adjustment for protein binding. Changes in protein binding for drugs that are highly bound to proteins can have a significant impact on safety and the pharmacologic activity.^15^ However, because gepotidacin has a low plasma protein binding (∼33%), a significant impact on safety or efficacy due to potential changes on protein binding in hepatic impairment is not expected. Therefore, the interpretation of the plasma PK is based on total drug exposure.

Overall, administration of 1500 mg of oral gepotidacin in this study was generally tolerated. In participants with normal hepatic function and participants with moderate and severe hepatic impairment, there were few AEs, and most were mild in intensity. The safety profile for gepotidacin in this study in participants with different stages of hepatic impairment was consistent with that observed in previous studies conducted in healthy participants.

The results of this study indicate that dose adjustments may not be needed in patients with mild to moderate hepatic impairment; however, in severe hepatic impairment an increase in the dosing interval or dose reduction may be required.

## Conflicts of Interest

M.H., C.T., A.B., and E.D. were employees of GlaxoSmithKline during the study conduct and hold company stock. T.C.M. and R.A.P. were the study investigators funded by GlaxoSmithKline during the conduct of the study. M.H. is currently at Agios Pharmaceuticals, Cambridge, Massachusetts. C.T. is employed by Gan and Lee Pharmaceuticals, Bridgewater, New Jersey. All authors meet the criteria for authorship set forth by the International Committee for Medical Journal Editors.

## Funding

Funding for this study was provided by GlaxoSmithKline (ClinicalTrials.gov identification number NCT03562117). This work was also supported in whole or in part with federal funds from the Office of the Assistant Secretary for Preparedness and Response, Biomedical Advanced Research and Development Authority, under Other Transaction Authority Agreement No. HHSO100201300011C. Editorial support (development of the first draft, assembling tables and figures, collating author comments, and referencing) was provided by Guissou Dabiri, PhD, and was funded by GlaxoSmithKline.

## Data Sharing Statement

Anonymized individual participant data and study documents can be requested for further research from www.clinicalstudydatarequest.com.

## Supporting information

Supporting Information.
