# Supplementary material for: Pharmacokinetics of Gepotidacin in Subjects With Normal Hepatic Function and Hepatic Impairment
Source: Clin Pharmacol Drug Dev. 2021 Jan 15;10(6):588–97. doi: 10.1002/cpdd.913 (PMC8248074; doi:10.1002/cpdd.913)
Supplement: Supplementary file 1 — Supporting Information. [file CPDD-10-588-s001.docx]

**Supplementary Material**

**Supp. Table 1 Analysis of Variance of Gepotidacin Plasma Pharmacokinetic Parameters by Hepatic Function**

| **Parameter** | **Hepatic Function Group** | **N** | **n** | **Geometric LS Means** | **Ratio of Geometric LS Means  (Relative to Normal)** | **90% CI of the Ratio** |
| --- | --- | --- | --- | --- | --- | --- |
| AUC_0-∞_ (μg•hr/mL) | Moderate | 8 | 8 | 19.5 | 1.23 | (0.86, 1.75) |
|  | Normal | 9 | 9 | 15.9 |  |  |
|  | Severe | 8 | 8 | 25.4 | 1.72 | (1.27, 2.33) |
|  | Normal | 8 | 8 | 14.8 |  |  |
| C_max_ (μg/mL) | Moderate | 8 | 8 | 3.91 | 1.22 | (0.69, 2.17) |
|  | Normal | 9 | 9 | 3.20 |  |  |
|  | Severe | 8 | 8 | 5.54 | 1.87 | (1.10, 3.18) |
|  | Normal | 8 | 8 | 2.96 |  |  |

CI = confidence interval; LS = least squares; N = Number of participants in the hepatic function group; n = number of participants with evaluable data

For AUC_0-∞_ and Cmax, analysis of variance with hepatic function group as a fixed effect was performed on the natural ln-transformed parameters.

**Supp. Table 2 Statistical Analysis of Gepotidacin Plasma Tmax**

| **Parameter** | **Hepatic Function Group** | **N** | **n** | **Median** | **Hepatic Function Group Comparison** | **Median Difference and 90% CIs of Median Difference** | **p-value** |
| --- | --- | --- | --- | --- | --- | --- | --- |
| Tmax (hr) | Moderate | 8 | 8 | 2.75 | Moderate-Normal | 0.00 (-1.00, 1.00) | 0.7916 |
|  | Normal | 9 | 9 | 3.00 |  |  |  |
|  | Severe | 8 | 8 | 2.25 | Severe-Normal | -1.00 (-2.00, 0.00) | 0.1277 |
|  | Normal | 8 | 8 | 3.00 |  |  |  |
| CI = confidence interval; N = Number of participants in the hepatic function group; n = number of participants with evaluable data  Median difference and Asymptotic (Moses) 90% CI of median difference was based on Hodges-Lehmann estimation. The p-value was based on Mann-Whitney U test (Wilcoxon rank sum test). | | | | | | | |

**Supp. Table 3 Analysis of Variance of Gepotidacin Urine Pharmacokinetic Parameters by Hepatic Function**

| **Parameter** | | **Hepatic Function Group** | **N** | **n** | **Geometric LS Means** | **Ratio of Geometric LS Means (Relative to Normal)^1^** | **90% CI of the Ratio** |
| --- | --- | --- | --- | --- | --- | --- | --- |
| AUC_0-48_ (µg•hr/mL) | | Moderate | 8 | 5 | 3162 | 3.19 | (1.35, 7.56) |
|  |  | Normal | 9 | 6 | 991 |  |  |
|  |  | Severe | 8 | 6 | 3902 | 3.94 | (1.67, 9.26) |
|  |  | Normal | 8 | 6 | 991 |  |  |
| CLr (L/hr) | | Moderate | 8 | 6 | 9.45 | 1.27 | (-1.84, 4.38) |
|  |  | Normal | 9 | 6 | 8.18 |  |  |
|  |  | Severe | 8 | 6 | 12.46 | 4.29 | (0.54, 8.03) |
|  |  | Normal | 8 | 6 | 8.18 |  |  |
| CI = confidence interval; LS = least squares; N = Number of participants in the hepatic function group; n = number of participants with evaluable data  Note: Analysis of variance with hepatic function group as a fixed effect was performed on the natural ln-transformed parameter AUC_0-48_ and non-transformed parameter CLr.  1. For non-transformed CLr, LS mean difference between hepatic impairment groups and normal hepatic function group, and 90% CIs for the difference are presented. PK parameters derived using urine concentrations from samples with protocol deviations (missed urine void for PK assessment and/or urine PK sample analyzed outside of established stability) were excluded from the statistical analysis. | | | | | | |  |

**Supp. Table 4 Analysis of Variance of Gepotidacin Saliva Pharmacokinetic Parameters by Hepatic Function**

| **Parameter** | **Hepatic Function Group** | **N** | **n** | **Geometric LS Means** | **Ratio of Geometric LS Means (Relative to Normal)** | **90% CI of the Ratio** | |
| --- | --- | --- | --- | --- | --- | --- | --- |
| AUC_0-∞_ (μg•hr/mL) | Moderate | 8 | 6 | 9.53 | 1.19 | (0.81, 1.73) | |
|  | Normal | 9 | 9 | 8.04 |  |  | |
|  | Severe | 8 | 8 | 14.28 | 1.77 | (1.21, 2.59) | |
|  | Normal | 8 | 8 | 8.05 |  |  | |
| Cmax (μg/mL) | Moderate | 8 | 8 | 1.65 | 1.28 | (0.80, 2.04) | |
|  | Normal | 9 | 9 | 1.29 |  |  | |
|  | Severe | 8 | 8 | 2.70 | 2.10 | (1.24, 3.56) | |
|  | Normal | 8 | 8 | 1.29 |  |  | |
| CI = confidence interval; LS = least squares; N = Number of participants in the hepatic function group; n = number of participants with evaluable data  For AUC_0-∞_ and Cmax, analysis of variance with hepatic function group as a fixed effect was performed on the natural ln-transformed parameters. | | | | | | |  |

**Supp. Table 5 Statistical Analysis of Gepotidacin Saliva Tmax**

| **Parameter** | **Hepatic Function Group** | **N** | **n** | **Median** | **Hepatic Function Group Comparison** | **Median Difference and 90% CIs of Median Difference** | **p-value** |
| --- | --- | --- | --- | --- | --- | --- | --- |
| Tmax (hr) | Moderate | 8 | 8 | 3.92 | Moderate-Normal | 0.500 (-0.500, 2.00) | 0.5544 |
|  | Normal | 9 | 9 | 3.00 |  |  |  |
|  | Severe | 8 | 8 | 3.00 | Severe-Normal | 0.00 (-1.00, 0.917) | 0.7116 |
|  | Normal | 8 | 8 | 3.46 |  |  |  |
| CI = confidence interval; N = Number of participants in the hepatic function group; n = number of participants with evaluable data  Note: Median difference and Asymptotic (Moses) 90% CI of median difference was based on Hodges-Lehmann estimation. The p-value was based on Mann-Whitney U test (Wilcoxon rank sum test). | | | | | | | |
